# MicroRNA-494 promotes cancer progression and targets adenomatous polyposis coli in colorectal cancer

**DOI:** 10.1186/s12943-017-0753-1

**Published:** 2018-01-05

**Authors:** Ying Zhang, Lu Guo, Yuhuan Li, Gui-Hai Feng, Fei Teng, Wei Li, Qi Zhou

**Affiliations:** 1 State Key Laboratory of Stem Cell and Reproductive Biology, Institute of Zoology, Chinese Academy of Sciences, Beijing, 100101 China; 2 University of Chinese Academy of Sciences, Beijing, 100049 China

**Keywords:** Colorectal cancer, microRNA, miR-494, Apc

## Abstract

**Background:**

Aberrant activation of the Wnt/β-catenin signaling pathway is frequently observed in colorectal cancer (CRC). β-catenin is the major Wnt signaling pathway effector and inactivation of adenomatous polyposis coli (APC) results in nuclear accumulation of β-catenin. It has been suggested that inactivation of APC plays an important role in activation of the Wnt/β-catenin pathway and in the progression of colorectal tumorigenesis. However, the mechanism through which APC mediates colorectal tumorigenesis is not understood. Increasing evidence suggests that the dysregulation of microRNAs (miRNAs) is involved in colorectal tumorigenesis. Although miR-494 has been reported as being an upregulated miRNA, the interplay between miR-494 and APC-mediated colorectal tumorigenesis progression remains unclear.

**Methods:**

The expression of miR-494 in tissues from patients diagnosed with CRC was analyzed using a microarray and real-time PCR. The effects of miR-494 on cell proliferation and tumorigenesis in CRC cells were analyzed by flow cytometry, colony formation assays, BrdU incorporation assays, and CCK8 assays. The correlation between miR-494 expression and APC expression, as well as the mechanisms by which miR-494 regulates APC in CRC were also addressed.

**Results:**

miR-494 was significantly upregulated in CRC tissues, and this increase was negatively associated with APC expression. APC was confirmed to be a direct target of miR-494 in CRC. Furthermore, overexpression of miR-494 induced Wnt/β-catenin signaling by targeting APC, thus promoting CRC cell growth.

**Conclusions:**

This study provides novel insights into the role of miR-494 in controlling CRC cell proliferation and tumorigenesis, and identifies miR-494 as a potential prognostic marker and therapeutic target.

## Background

Colorectal cancer (CRC) is one of the most common malignancies in the world, with over one million new patients diagnosed every year [1]. CRC progression is accompanied by the accumulation of mutations in tumor-suppressor genes and oncogenes, including *adenomatous polyposis coli (APC)* [2]. Inactivation of *APC* is a major initiating event in colorectal tumorigenesis [3]. Specifically, mutations in *APC* are a leading cause of CRC [4]. Mutations in *APC* have been found in all patients diagnosed with familial adenomatous polyposis, as well as in almost 90% of patients diagnosed with CRC [5]. Most of the mutations in *APC* generate premature stop codons leading to truncated proteins that lack β-catenin binding sites. APC-free β-catenin stimulates the Wnt signaling pathway, leading to the active transcription of target genes such as c-Myc and cyclin D1, thereby promoting tumorigenesis [6, 7].

APC and Axin serve as essential scaffolds for glycogen synthase kinase 3 beta (GSK-3β) and β-catenin, and impaired association of APC, Axin, with β-catenin leads to constitutive activation of the Wnt signaling pathway [8, 9]. In the intestine, the canonical Wnt pathway maintains the proliferative cell layer in the crypts [10]. Upon activation of the Wnt pathway, β-catenin is released from the cytoplasmic complex formed by APC, Axin, and GSK-3β. Consequently, β-catenin is then able to bind the T-cell factor/lymphoid enhancer factor binding factor (TCF/LEF) transcription factors, resulting in increased transcription of downstream targets such as c-Myc or cyclin D1. In contrast, in differentiated intestinal epithelial cells, APC acts as a negative regulator of the Wnt signaling pathway by binding to β-catenin in order to induce its degradation [11].

MicroRNAs (miRNAs), are a class of naturally occurring small, noncoding RNAs comprising of 19 to 25 nucleotides, that are an important class of cellular regulators that modulate gene expression, and thereby influence cell fate and function [12–16]. miRNAs function by binding to target mRNAs via sequence complementarity, and repress translation or induce degradation of their target mRNAs [17, 18]. So far, a number of miRNAs have been ascribed oncogenic or tumor-suppressive functions, and they are involved in almost every type of cancer, including breast, lung, gastric carcinoma, and CRC [19–22]. Some miRNAs have been studied for their roles in colorectal carcinogenesis [23–26]. For example, miR-494 has previously been reported to be upregulated in CRC, and it promotes cell migration and invasion in CRC by directly targeting phosphatase and tensin homolog (PTEN) [27]. The roles and potential mechanisms of miRNAs, mediated by APC, in CRC are still largely unknown.

Here, we report that miR-494 activates the Wnt/β-catenin signaling pathway by suppressing the expression of APC and consequently plays an important role in the development and progression of CRC.

## Methods

### Tissue specimens and immunohistochemistry (IHC) staining

The Ethics Committee of Institute of Zoology approved this study, and all patients gave their informed consent prior to surgery. Colon carcinoma tissues from human patients were obtained from Beijing 301 Military General Hospital (Beijing, China). The patients clinical characteristics are shown in Table 1. Detailed information including demography, clinical characteristics, histopathology, APC mutation status, and survival status were collected for all patients. Patients were followed after surgical treatment with a median follow-up of 61 months (range, 8–122 months). All patients were staged based on the Tumor-Node-Metastasis (TNM) classification. For mRNA extraction, samples were frozen in liquid nitrogen immediately after surgical removal and maintained at −80 °C until use. Additional samples were fixed in 10% neutral-buffered formalin overnight, processed, paraffin embedded, and sectioned. A patient tissue with one synonymous mutation in the codon encoding amino acid 1828 in APC, that did not cause a change in amino acid sequence, was selected for IHC analysis. IHC analysis was performed using 3 μm sections which were incubated with an anti-APC antibody (Abcam catalog no. 154906) overnight at 41 °C in a humidified chamber, followed by incubation with an HRP-conjugated secondary antibody for 2 h. Staining was completed after 5 to 10 min incubation with 3,3′-diaminobenzidine (DAB) as substrate, which resulted in a brown-colored precipitate at the antigen site.

**Table 1:** Clinical correlation between miR-494 expression and other clinicopathological characteristics in CRC

Characteristics	N of cases	High miR-494 group	*P*-value^a^
Age (years)	46	20	0.476
≤ 60	24	11	
> 60	22	9	
Sex			0.887
Men	28	12	
Women	18	8	
TNM stage			*0.004
I + II	19	6	
III + IV	27	14	
Differentiation			*0.034
Well	35	14	
Poorly	11	6	
APC mutation status			0.567
Wild-type	5	2	
Mutant	41	18	
Survival status			0.476
Dead	22	9	
Alive	24	11	

^a^Chi-square test, *P < 0.05. CRC, colorectal carcinoma

### Cell lines and cell culture

Human colon cancer cell lines were obtained from American Type Culture Collection (Manassas, VA). HCT-116 and HT-29 cells were maintained in Dulbecco’s modified Eagle’s medium (DMEM) supplemented with 10% fetal bovine serum (FBS). Cells were maintained in a humidified incubator equilibrated with 5% CO_2_ at 37 °C.

### Microarray analysis

Total RNA was extracted from 17 tumor tissues and paired normal colorectal tissues using TRIzol reagent (Invitrogen). miRNA microarray profiling was performed as previously described [28]. Data analysis was performed using GeneSpring GX software (Agilent). An miRNA was designated as overexpressed if the expression in tumor tissues was greater than 2.0-fold that in normal colorectal epithelial tissues.

### Real-time PCR detection of mature miRNAs

Total RNA was isolated using TRIzol reagent (Invitrogen) according to the manufacturer’s protocol. All-in-One™ miRNA First-Strand cDNA Synthesis Kit (Genecopoeia) was used to transcribe 10 μL of purified RNA. Real-time PCR, using miR-specific primers and universal adaptor PCR primers (Genecopoeia), was performed with a Stratagene Mx3000P QPCR System (Genetimes Technology, Shanghai, China). The reactions were incubated in a 96 well plate at 95 °C for 10 min, followed by 40 cycles at 95 °C for 15 s, and 60 °C for 1 min. All reactions were run in triplicate.

### RNA extraction and real-time PCR

Total RNA was isolated as described above. For reverse transcription into cDNA, 1 μg of RNA from each sample was incubated with random primers and then subjected to real-time PCR. Real-time quantification was performed using a Stratagene Mx3000P quantitative PCR system (Genetimes Technology, Shanghai, China). The reaction solutions were incubated in a 96-well plate at 95 °C for 10 min, followed by 40 cycles at 95 °C for 15 s, and 60 °C for 1 min. All reactions were run in triplicate. The primer pairs used were as follows: APC, 5′-CTGCGGACCGAGGTTGGCTC-3′ (forward) and 5′-CTTCCTGCCAGACGCTCGCC-3′ (reverse); and GAPDH, 5′-CTCTGCTCCTCCTGTTCGAC-3′ (forward) and 5′-CGACCAAATCCGTTGACTCC-3′ (reverse).

### Oligonucleotide transfections

All commercial miRNAs and the APC targeted small interfering RNAs (siRNAs) were synthesized and purified by RiboBio Co. (Guangzhou, China). The APC siRNA sequences used were APC-siRNA1, 5′-GGATCAGCCTATTGATTAT-3′ and APC-siRNA2, 5′-GTACGCCAGTCAACTTTCA-3′. Transfections of the aforementioned miRNAs and siRNAs were performed using Lipofectamine 2000 (Invitrogen), according to the manufacturer’s instructions.

### Western blot analysis

Western blotting was performed as previously described (31). The commercial antibodies used were anti-APC (Abcam catalog no. 154906), anti-β-catenin (Santa Cruz catalog no. 7963), anti-cyclin D1 (Cell Signaling Technology catalog no. 2978S), and anti-fibrillarin (Abcam catalog no. ab5812).

### Colony formation assays

HCT-116 cells were transfected with the indicated long-lasting synthetic miRNAs. The commercial miRNAs used were a negative miR-control (Ribobio catalog no. miR04101–1-10), a miR-494 mimic (Ribobio catalog no. miR40002816–1-10), a negative anti-miR-control (Ribobio catalog no. miR03101–1-10), or an anti-miR-494 (Ribobio catalog no. miR30002816–1-10). At 48 h post-transfection, cells were plated in triplicate at 500 cells per 60 mm dish. After 7 days, the colonies were stained with 0.1% crystal violet solution and counted using a light microscope.

### Flow cytometry

Cells were harvested by trypsinization, washed with ice-cold PBS, and fixed in 70% ice-cold ethanol in PBS. Before staining, cells were sedimented in a chilled centrifuge and resuspended in cold PBS. Bovine pancreatic RNase (Sigma–Aldrich) was added to a final concentration of 2 μg/mL, and the cells were incubated at 37 °C for 30 min, followed by incubation with 20 μg/mL propidium iodide (Sigma–Aldrich) for 20 min at room temperature. The cell cycle profiles of 2 × 10^4^ cells were analyzed using a FACSCalibur flow cytometer (BD Biosciences).

### Statistical analysis

All experiments were repeated at least three times. Data shown are presented as mean ± SD of three or more independent experiments. A Student’s *t*-test was used for statistical analysis of data. Differences in clinicopathological characteristics between the two groups were examined by Fisher’s and chi-square tests. All *P*-values were determined using two-sided tests and statistical significance was based on a *P*-value of 0.05. Correlation analysis was performed using scatter plots and Pearson’s correlation analysis. The correlation coefficients and the corresponding *P*-values were calculated by the cor.test function of R.

## Results

### miR-494 is overexpressed in colorectal cancer

**Fig. 1:**
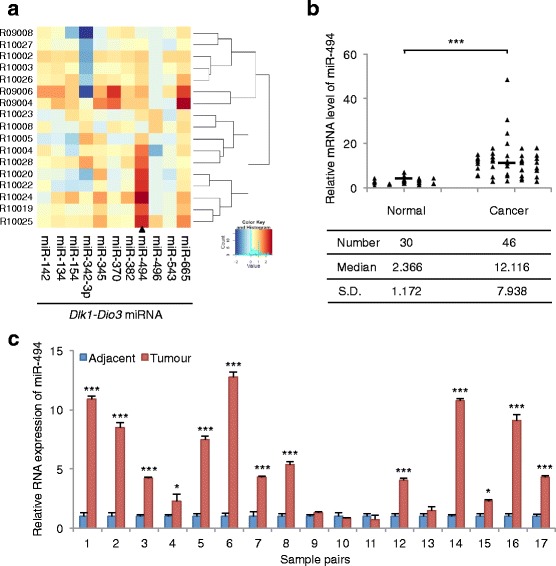
miR-494 is overexpressed in CRC. **a** Hierarchical clustering of miRNA expression profiles from the *Dlk1-Dio3* region obtained from 17 pairs of CRC tissues compared with adjacent, non-tumor tissues. **b** The miR-494 expression levels in CRC tissues and non-tumor tissues, evaluated by real-time PCR. **c** The expression levels of miRNA-494 in paired samples of CRC tissues and matched adjacent non-tumor tissues. (*, *P* < 0.05; ***, *P* < 0.001, Student’s *t*-test)

The *Dlk1-Dio3* region is located on human chromosome 14q32, and constitutes one of the largest miRNA clusters in the human genome [29]. Many of these miRNAs are differentially expressed in various cancers, as well as several other disease states [30]. The expression and roles of *Dlk1-Dio3* region miRNAs in oncogenesis are very controversial and differ in different tissues [30]. We examined several miRNAs from the *Dlk1-Dio3* region using a microarray-based expression analysis of 17 CRC tissues and matched non-tumor adjacent tissues. As shown in Fig. 1a, a comparative analysis indicated that miR-494 was differentially overexpressed in CRC tissue when compared to that in adjacent, non-tumor tissue from the same patient. We further conducted real-time PCR to analyze the expression of miR-494 in 30 non-tumor adjacent tissues and 46 carcinoma tissues from CRC patients (Fig. 1b), as well as in the 17 pairs of carcinoma tissues and matched non-tumor adjacent tissues (Fig. 1c). The real-time PCR data was consistent with the microarray data. Taken together, these results indicate that miR-494 is upregulated in CRC.

### Upregulation of miR-494 is associated with advanced clinicopathological features of CRC

To determine the clinical significance of miR-494 in CRC, we analyzed the association between miR-494 CRC tissue expression levels and various CRC clinicopathological parameters. The median miR-494 expression in all 46 CRC patients was 12.116. Using the median expression level as a cutoff, the patients were divided into two groups, a high miR-494 expression group (*n* = 20), and a low miR-494 expression group (*n* = 26). As shown in Table 1, miR-494 was significantly upregulated in CRC patients with a higher TNM stage (*P* = 0.004). We also found that there were increased miR-494 levels in poorly differentiated CRC tissue compared to well-differentiated CRC tissue (*P* = 0.034). However, miR-494 expression was not significantly correlated with age, gender, mutation in APC, or survival status.

### miR-494 promotes CRC cell proliferation

**Fig. 2:**
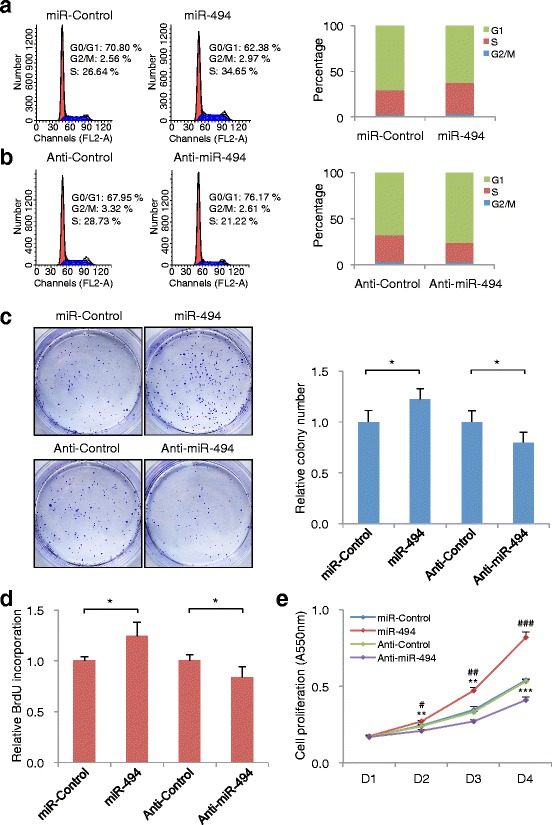
miR-494 promotes the proliferation of CRC cells. **a** The effect of miR-494 overexpression on cell cycle progression. HCT-116 cells were transfected with a control miRNA or a miR-494 mimic for 24 h and then switched to conditioned medium without serum for 24 h. The cells were cultured in medium containing 10% FBS for 24 h and collected for cell cycle analysis by flow cytometry. The data shown in the right-hand panel are representative of three independent experiments. Representative profiles are shown in the left-hand panel. **b** The effect of miR-494 knockdown on cell cycle progression. After HCT-116 cells were transfected with the control miRNA inhibitor or the miR-494 inhibitor for 24 h, cells were switched to conditioned medium without serum for 24 h. The cells were then cultured in medium containing 10% FBS for 24 h and collected for cell cycle analysis by flow cytometry. The data shown in the right-hand panel are representative of three independent experiments. Representative profiles are shown in the left-hand panel. **c** Colony formation assay. HCT-116 cells stably expressing the indicated miRNAs were maintained in culture media for 7 days and stained with crystal violet. The number of colonies in each condition was counted, and is expressed as a mean ± S.D. from triplicate experiments. *, *P* < 0.05 (Student’s *t*-test). **d** The effect of miR-494 on cell proliferation. HCT-116 cells were transfected with the indicated constructs, and cell proliferation was measured by BrdU incorporation. Data are mean ± S.D. from triplicate experiments. *, *P* < 0.05 (Student’s *t*-test). **e** The effect of miR-494 on cell proliferation. HCT-116 cells were transfected with a miR-494 mimic or with a miR-494 inhibitor. CCK8 assays were performed at 1, 2, 3, and 4 days post-transfection. Data are mean ± S.D. from triplicate experiments. **, *P* < 0.01;***, *P* < 0.001, compared to Anti-Control. #, *P* < 0.05; ##, *P* < 0.01; ###, *P* < 0.001, compared to miR-Control, Student’s *t*-test

To explore the role of miR-494 upregulation in the development and progression of CRC, we examined its effects on cellular proliferation and tumorigenesis. The HCT-116 line was chosen as it expresses wild-type APC, and it is widely used in CRC research. Flow cytometry analysis revealed that overexpression of a miR-494 mimic caused an increase in the percentage of cells in S phase, and a decrease in the percentage of cells in the G1/G0 phase. Conversely, suppression of miR-494 expression using a miR-494 inhibitor increased the percentage of cells in the G1/G0 phase (Fig. 2a and b). To investigate the role of miR-494 in colon tumorigenesis, a colony formation assay was performed, which revealed that miR-494 overexpression significantly increased the proliferation rate of CRC cells (Fig. 2c). We further examined the effects of suppressing miR-494 expression on CRC cell proliferation. Consistent with the above-mentioned results, a colony formation assay showed that suppression of miR-494 expression dramatically inhibited the growth rate of HCT-116 CRC cells, as compared to control cells transfected with a control miRNA (Fig. 2c). To further demonstrate the importance of miR-494 in cell proliferation, we performed a BrdU incorporation assay, which is a more sensitive assay to measure cell proliferation. As a result, the number of BrdU-positive cells increased when miR-494 was overexpressed, whereas the number decreased when miR-494 was downregulated (Fig. 2d). A second cell proliferation study, conducted using a CCK8 assay, also indicated a role for miR-494 in positively regulating cell proliferation (Fig. 2e). Collectively, our data suggest that miR-494 may mediate CRC cell proliferation by regulating the G1/S transition.

### miR-494 directly targets APC in CRC cells

**Fig. 3:**
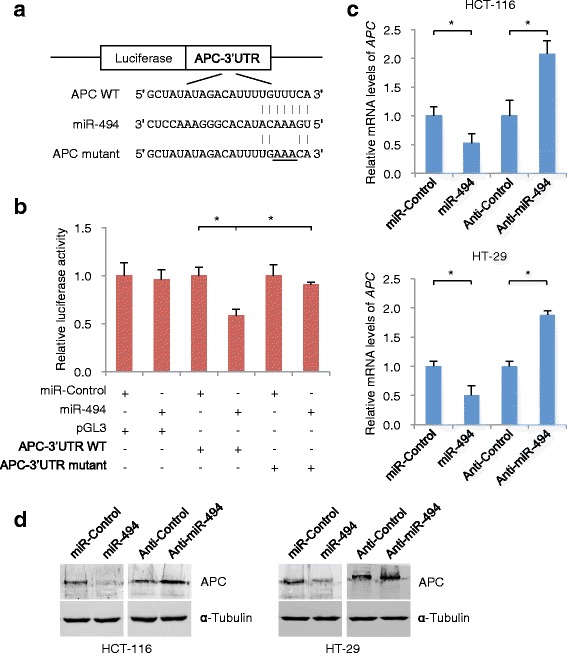
Luciferase reporter assays demonstrating the target relationship between miR-494 and APC mRNA. **a** Predicted miRNA binding sites within the 3′-UTR of APC mRNA. The pairing between miR-494 and the putative binding sites in the 3′-UTR of APC mRNA are shown. Mutations in the APC 3′-UTR are underlined. **b** Luciferase activity, normalized to renilla luciferase activity, was measured in homogenates of HEK293T cells transfected with the wild-type 3′-UTR and mutant 3′-UTR APC luciferase constructs and a miR-494 mimic or a scrambled miRNA control. **c** APC mRNA expression is reduced by the miR-494 mimic and increased by the miR-494 inhibitor. **d** Expression of APC protein is reduced by the miR-494 mimic and increased by the miR-494 inhibitor. (*, *P* < 0.05, Student’s *t*-test, *n* = 3 independent experiments)

Using a bioinformatics analysis, we identified putative binding sites for miR-494 within the 3′-UTR of the human APC mRNA (Fig. 3a). To validate APC as a miR-494 target, we constructed a luciferase expression vector containing the 3′-UTR segment of APC along with the putative miR-494 binding sites. Co-transfection of a miR-494 mimic and the APC 3′-UTR expression vector into HEK293T cells resulted in a significant suppression of APC luciferase activity. In addition, mutating the putative miR-494 binding sites completely eliminated this inhibitory effect (Fig. 3b). These data strongly suggest that APC is indeed the direct target of miR-494.

To examine whether miR-494 can directly target the APC mRNA in HCT-116 and HT-29 CRC cell lines, we transiently transfected the miR-494 mimic into HCT-116 and HT-29 cells. Endogenous APC mRNA and APC protein levels were then measured 48 h after transfection. As a result, the expression of both APC mRNA and APC protein decreased in the presence of the miR-494 mimic (Fig. 3c and d). In contrast, when HCT-116 and HT-29 cells were transfected with the miR-494 inhibitor, the APC mRNA and APC protein levels increased (Fig. 3c and d).

### Downregulation of APC is inversely correlated with miR-494 expression in CRC

**Fig. 4:**
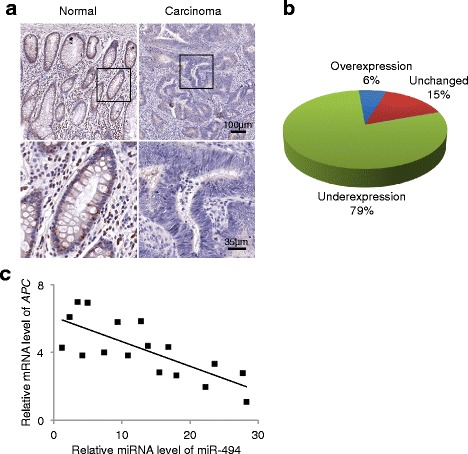
Expression of APC is often decreased and inversely correlated with miR-494 expression in human CRC tissues. **a** Immunohistochemical staining of APC in tumor tissue and corresponding normal colonic epithelium. Normal epithelium shows vesicular brown cytoplasmic APC staining. This staining is nearly absent in the tumor tissue. **b** APC mRNA expression is frequently decreased in tumor tissues when compared to the matched non-cancerous tissues, as evaluated by real-time PCR. **c** The relative levels of miR-494 expression were plotted against the relative levels of APC mRNA expression

To further evaluate the relationship between miR-494 and APC in human CRC, we analyzed the expression of APC and miR-494 in paired CRC and non-tumor adjacent tissue using immunohistochemistry and real-time PCR. Using both immunohistochemistry (Fig. 4a) and real-time PCR (Fig. 4b), tumors showed decreased APC expression when compared with expression in tumor-adjacent tissues. Furthermore, using real-time PCR, APC mRNA expression levels in tumor tissues were found to be inversely correlated with miR-494 levels (Fig 4c). This relationship had a Pearson’s correlation coefficient of −0.5679 (*P* < 0.0001), indicating a strong negative correlation between miR-494 and APC expression levels in colon carcinomas. Taken together, these data suggest that decreased APC expression may result from overexpression of miR-494 in human CRC.

### miR-494 activates the Wnt/β-catenin pathway

**Fig. 5:**
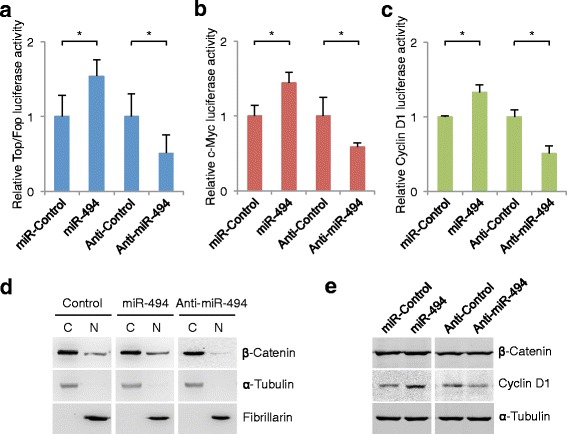
miR-494 induces β-catenin signaling (Top/Fop, c-Myc, cyclin D1). **a** Dual luciferase assay demonstrating the effect on Top/Fop reporter activity in HCT-116 cells. Values were normalized to a renilla transfection control. The figure shown is a representative of at least three independent experiments. **b** Dual luciferase assay showing the effect of expression of a miR-494 mimic on a c-Myc reporter construct in HCT-116 cells. **c** Dual luciferase assay showing the effect of expression of a miR-494 mimic on a cyclin D1 reporter construct in HCT-116 cells. **d** Western blot analysis of β-catenin expression in the cytoplasm (C) and nucleus (N) of the indicated cells. α-tubulin and fibrillarin were measured by western blot to monitor the efficiency in the preparation of cytosolic and nuclear protein extracts, respectively. **e** Western blot analysis of β-catenin and cyclin D1 in the indicated cells. (*, *P* < 0.05, Student’s *t*-test, *n* = 3 independent experiments)

Next, we investigated the functional relevance of the interaction between miR-494 and APC by determining the effects of changes in their expression levels on the activity of the Wnt pathway. First, we examined the effects of changes in miR-494 expression levels on the transcriptional activity of the TCF transcription factor. HCT-116 cells were transfected with a luciferase reporter construct harboring either three optimal TCF-binding sites (Topflash) or three mutated TCF-binding sites (Fopflash). Transfection with the miR-494 mimic significantly increased Top/Fop transcriptional activity, whereas transfection with the miR-494 inhibitor significantly decreased Top/Fop transcriptional activity (Fig. 5a). Next, we examined the induction of c-Myc and cyclin D1 activity, important downstream target genes in the Wnt/β-catenin pathway (35). We used luciferase reporter constructs under the control of promoters containing either of four Myc or cyclin D1 responsive elements. We observed an increase in both c-Myc and cyclin D1 reporter activities in cells co-transfected with the miR-494 mimic, and a corresponding decrease in c-Myc and cyclin D1 reporter activity in cells co-transfected with the miR-494 inhibitor (Fig. 5b and c). Furthermore, cellular fractionation showed that miR-494 mimic overexpression promoted the nuclear accumulation of β-catenin (Fig. 5d), indicating that miR-494 activates the Wnt/β-catenin signaling pathway by promoting nuclear β-catenin accumulation. Moreover, we observed an increase in the cyclin D1 protein, a Wnt/β-catenin signaling pathway target gene, in cells overexpressing the miR-494 mimic (Fig. 5e). Thus, overexpression of miR-494 activates the Wnt/β-catenin pathway.

### Suppression of APC is functionally important for the biological effects of miR-494

**Fig. 6:**
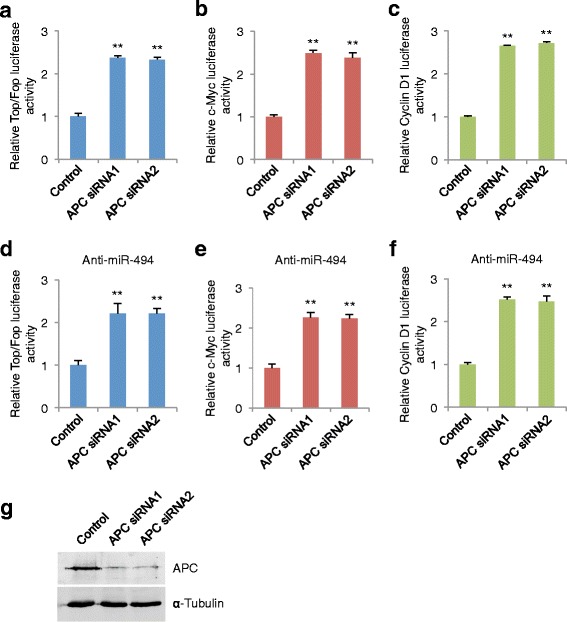
APC is functionally involved in miR-494–induced β-catenin signaling activity of CRC cells. **a** Dual luciferase experiment demonstrating the effects on Top/Fop reporter activity in the indicated cells. Values were normalized to a renilla transfection control. The figure shown is representative of at least three independent experiments. **b** Dual luciferase assay showing the effects on a c-Myc reporter construct in the indicated cells. **c** Dual luciferase assay showing the effects on a cyclin D1 reporter construct in the indicated cells. **d** Dual luciferase experiment demonstrating the effects on Top/Fop reporter activity in the indicated cells following miR-494 downregulation. **e** Dual luciferase assay showing the effects on a c-Myc reporter construct in the indicated cells following miR-494 downregulation. **f** Dual luciferase assay showing the effects on a cyclin D1 reporter construct in the indicated cells following miR-494 downregulation. **g** APC western blot of the indicated cells. (**, *P* < 0.01, Student’s *t*-test, n = 3 independent experiments)

To explore the functional significance of APC depletion in upregulating the Wnt/β-catenin pathway in colon cancer cell lines, as well as in the activation of β-catenin induced by miR-494, we studied the effects of APC depletion using specific siRNAs. As shown in Fig. 6a, b, and c, siRNA silencing of APC expression enhanced the Top/Fop, c-Myc, and cyclin D1 transcriptional activities. In addition, silencing APC also increased Top/Fop, c-Myc and cyclin D1 transcriptional activities in HCT-116 cells whose miR-494 expression levels were suppressed (Fig. 6d, e, and f). These results demonstrate that APC lies downstream of miR-494 and is functionally important for miR-494–induced Wnt/β-catenin signaling in colon cancer cell lines.

## Discussion

The key finding in this study is that miR-494 expression is markedly upregulated in CRC tissues compared to normal colorectal tissues. Furthermore, ectopic expression of miR-494 enhances the proliferation of CRC cells, whereas inhibition of miR-494 expression has the opposite effect. Moreover, we demonstrate that miR-494 upregulation in CRC cells activates the Wnt/β-catenin pathway by directly targeting APC. These findings suggest that this dysregulation of miR-494 levels may play an important role in promoting proliferation and tumorigenesis in CRC.

Aberrant Wnt/β-catenin signaling has been linked to the pathogenesis of various diseases, including cancer, and is thought to promote tumor progression [23–28]. Multiple key negative regulators, such as APC, GSK-3β, and Axin, are suppressed in cancer, contributing to the promotion of tumor progression through regulation of the Wnt/β-catenin pathway. For instance, inactivation of GSK-3β via kinases promotes mammary tumorigenesis by activation of the canonical Wnt pathway [31]. In mouse models, loss of APC function has been shown to lead to colorectal tumorigenesis through hyperactivation of Wnt/β-catenin signaling [32]. Herein, we demonstrate that miR-494 suppresses APC expression by directly targeting the 3′-UTR of the APC mRNA. Consistent with the tumor-suppressive effects of APC, miR-494 was upregulated in CRC, and its overexpression dramatically promoted CRC cell proliferation.

The Wnt/β-catenin signaling pathway is controlled at multiple levels. β-catenin is the major cellular effector of Wnt signaling and is normally retained by a degradation complex containing Axin, APC, GSK-3β, and casein kinase 1a. In the absence of Wnt, the complexed β-catenin is targeted for proteasome-mediated degradation following its phosphorylation and ubiquitination [33–35]. APC is crucial for the capture of β-catenin by the degradation complex, and binds β-catenin directly via its central armadillo repeats [36]. Upon Wnt activation, Axin is removed from the destruction complex allowing the release of β-catenin. The accumulated β-catenin enters the nucleus and binds to the TCF/LEF family of transcription factors to induce target gene expression [37, 38]. A key event therefore in both Wnt signal transduction and cancer cell proliferation is the regulation of β-catenin stability and activity. In this study, we have uncovered a mechanism for controlling APC expression, and thereby the activity of the Wnt pathway, through the action of miR-494, and suggest that miR-494 contributes to the pathogenesis of CRC.

miR-494 is a product of the *Dlk1-Dio3* region, which is located on human chromosome 14q32, and constitutes one of the largest miRNA clusters in the human genome. The expression patterns of miR-494 are not consistent across different types of tumors, suggesting that there is tissue-dependent regulation of expression of this miRNA, among other potential mechanisms [39–44]. In this study, we demonstrated that miR-494 is upregulated in CRC tissues compared to adjacent, non-cancerous tissues from the same patient. Furthermore, we demonstrated that ectopic miR-494 expression increases the growth rate of HCT-116 cells compared to a control miRNA, whereas suppression of miR-494 expression inhibited cell proliferation and colony formation. The data strongly suggest that upregulation of miR-494 correlates with clinical CRC progression and that miR-494 may function as an onco-miRNA.

## Conclusions

Our study has shown that elevated expression of miR-494 promotes cell proliferation and tumorigenesis in CRC by suppressing the expression of APC, an inhibitor of β-catenin signaling. These findings uncover a novel molecular mechanism for the hyperactivation of the Wnt/β-catenin signaling pathway in CRC, suggesting that miR-494 may serve as a potential therapeutic target for the treatment of CRC.

## Abbreviations

APC: Adenomatous polyposis coli
CRC: Colorectal cancer
DAB: Diaminobenzidine
DMEM: Dulbecco’s modified Eagle’s medium
FBS: Fetal bovine serum
GSK-3β: Glycogen synthase kinase 3 beta
IHC: Immunohistochemistry
miRNAs: MicroRNAs
PTEN: Phosphatase and tensin homolog
TCF/LEF: T-cell factor/lymphoid enhancer binding factor
TNM: Tumor-Node-Metastasis
